# A constrained maximum likelihood approach to developing well-calibrated models for predicting binary outcomes

**DOI:** 10.1007/s10985-024-09628-9

**Published:** 2024-05-08

**Authors:** Yaqi Cao, Weidong Ma, Ge Zhao, Anne Marie McCarthy, Jinbo Chen

**Affiliations:** 1 Department of Statistics, School of Science, Minzu University of China, Beijing, China; 2 Department of Biostatistics, Epidemiology and Informatics, Perelman School of Medicine, University of Pennsylvania, Philadelphia, PA 19104 USA; 3 Department of Mathematics and Statistics, Portland State University, Portland, PA 97201 USA

**Keywords:** Calibration, Constrained maximum likelihood estimation, Logistic regression, Risk prediction

## Abstract

The added value of candidate predictors for risk modeling is routinely evaluated by comparing the performance of models with or without including candidate predictors. Such comparison is most meaningful when the estimated risk by the two models are both unbiased in the target population. Very often data for candidate predictors are sourced from nonrepresentative convenience samples. Updating the base model using the study data without acknowledging the discrepancy between the underlying distribution of the study data and that in the target population can lead to biased risk estimates and therefore an unfair evaluation of candidate predictors. To address this issue assuming access to a well-calibrated base model, we propose a semiparametric method for model fitting that enforces good calibration. The central idea is to calibrate the fitted model against the base model by enforcing suitable constraints in maximizing the likelihood function. This approach enables unbiased assessment of model improvement offered by candidate predictors without requiring a representative sample from the target population, thus overcoming a significant practical challenge. We study theoretical properties for model parameter estimates, and demonstrate improvement in model calibration via extensive simulation studies. Finally, we apply the proposed method to data extracted from Penn Medicine Biobank to inform the added value of breast density for breast cancer risk assessment in the Caucasian woman population.

## Introduction

To evaluate the value of new predictors for improving risk assessment, the standard approach is to compare the performance of models with or without including the new predictors. However, this practice assumes that the study sample is representative of the target population of prediction. When data for new predictors is obtained from convenience samples, there may be differences in the distribution of risk predictors, outcome prevalence, and the relationship between outcomes and predictors when compared to the target population (Debray et al. 2015; Steyerberg 2019). Ignoring such discrepancies could lead to biased evaluations of the usefulness of these new predictors. For example, the evaluation of breast imaging biomarkers and polygenic risk scores for breast cancer risk assessment in Penn Biobank may not accurately inform the value of these new predictors in the U.S. Caucasian woman population.

To ensure proper performance comparison in the target population, ideally, the models should be calibrated to eliminate possible bias in risk estimates when the training data may follow a different distribution (Dalton 2013; Vergouwe et al. 2010; Ankerst et al. 2016; Pfeiffer et al. 2022). When the risk estimates are used to identify individuals at high risk, it is important to ensure calibration in the upper tail of the risk distribution (Song et al. 2015). However, the availability of independent testing data from the target population is often limited, posing a significant challenge for model evaluation and comparison. When the goal of model comparison is to inform the added value of new predictors, often the base model with conventional predictors has been extensively validated in the target population. For example, the Breast Cancer Risk Assessment Tool (“BCRAT”; Gail et al. 1989) was validated in multiple cohorts for projecting individualized risk for the U.S. Caucasian women (Bondy et al. 1994; Costantino et al. 1999; Rockhill et al. 2001). To evaluate the potential improvement that breast imaging biomarkers and polygenic risk score can make on BCRAT, the data made available from Penn Medicine Biobank tends to have stronger family history.

In this work, assuming access to a well-calibrated base model with only conventional predictors, we develop a novel semiparametric method for fitting a logistic regression model for predicting binary outcomes that include both conventional and new risk predictors. A key feature of our approach is that we allow the distribution of the study data to differ from that in the target population, while ensuring that the resulting model exhibits a similar level of calibration as the base model. Our method therefore facilitates proper evaluation on the added value of new predictors, but bypasses the need of independent validation sample. The effectiveness of our method relies on two important requirements. Firstly, we assume that the distribution of conventional risk predictors is known in the target population. Secondly, we require that the relationship between the new and conventional predictors remains identical in both the study and target populations. These assumptions ensure the feasibility of our method and pave the way for accurate model evaluation without relying on an independent validation dataset.

The central idea of the proposed method is to calibrate the fitted model against the base model by enforcing suitable constraints in maximizing the likelihood function. Since the base model is well-calibrated in the target population, the imposed constraints are constructed to ensure that the predicted risk by the fitted model is also reasonably unbiased. This work is closely related to recent literature on utilizing summary level information to enhance the statistical efficiency in estimating regression parameters (Chatterjee et al. 2016; Zheng et al. 2022a, b; Zhai and Han 2022). For instance, in the context of fitting a logistic regression model, a novel constrained semiparametric maximum likelihood approach (Chatterjee et al. 2016) leveraged an established regression relationship between the outcome and a subset of covariates, resulting in improved efficiency when estimating odds ratio association parameters. However, these methods assume a correctly specified model for the relationship between the outcome and covariates, and they also require the probability distribution of the outcome and covariates to be identical in both populations. To improve calibration of models for predicting time-to-event outcomes, a constrained empirical likelihood method (Zheng et al. 2022a, b) was recently proposed to adjust the discrepancy in baseline hazard rates, assuming that the study source and target populations share the same hazard ratio parameters. Notably, these works require randomly sampled data from the target population, and the external information was leveraged to increase statistical efficiency (Zhai and Han 2022). In contrast, our method specifically aims to reduce bias in risk estimation when the study sample is not representative of the target population.

The rest of the article is organized as follows. We present a constrained maximum likelihood (“cML”) method in Sect. 2 and study its theoretical properties, with technical details provided in Appendix. In Sect. 3, we apply cML method to a dataset assembled from Penn Medicine Biobank to assess whether percent mammographic density can potentially improve prediction of 5-year breast cancer risk in the U.S. Caucasian women. We report results from extensive simulation studies in Sect. 4. Some discussions are given in Sect. 5.

## Method

### Notation and likelihood function

Let *Y* denote the binary outcome of interest ($Y=1$: case; $Y=0$: control), $\varvec{X}= \left( X_{1},\ldots , X_p\right) ^{T}$ denote the *p* dimensional conventional risk predictors, and $\varvec{Z}= \left( Z_{1},\ldots , Z_q \right) ^{T}$ denote the *q* dimensional new candidate predictors. Data for $(Y, \varvec{X}, \varvec{Z})$ is observed for a random sample of *N* subjects, $(Y_{i},\varvec{X}_{i}^T,\varvec{Z}_{i}^T)$, $i=1\ldots N$, selected from a population $\mathcal {P}_{\text {S}}$, where the subscript “S” indicates “the study source population”. The probability and expectation of random variables on $\mathcal {P}_{\text {S}}$ are denoted as ${\text {Pr}}_{\text {S}}$ and $E_{\text {S}}$, respectively. Denote the target population of prediction and corresponding probability and expectation as $\mathcal {P}$, ${\text {Pr}}$ and *E*. The two populations $\mathcal {P}$ and $\mathcal {P}_{\text {S}}$ may be different but are related. For example, Caucasian woman patients in Penn Medicine Biobank ($\mathcal {P}_{\text {S}}$) represent a subset of the U.S. Caucasian woman population ($\mathcal {P}$). Let $\varphi (\varvec{X})$ denote the base model with conventional risk predictors $\varvec{X}$, which is well-calibrated in population $\mathcal {P}$ and has been evaluated across strata defined by $\varvec{X}$.

Our goal is to develop a well-calibrated model, $P(Y=1|\varvec{X}, \varvec{Z})$, for predicting the risk of *Y* using both conventional risk predictors, $\varvec{X}$, and new candidate predictors, $\varvec{Z}$, within the target population $\mathcal {P}$. The probability distribution of $\varvec{X}$ in $\mathcal {P}$, $\delta \left( {\textbf{x}}\right) \equiv {\text {Pr}}(\varvec{X}={\textbf{x}})$, is known from external sources. For example, $\delta \left( {\textbf{x}}\right)$ for conventional risk predictors in BCRAT can be estimated from the National Health Interview Survey. We allow difference between $\delta \left( {\textbf{x}}\right)$ and the distribution of $\varvec{X}$ in the source population, denoted as $\pi \left( {\textbf{x}}\right) \equiv {\text {Pr}}_{S}(\varvec{X}={\textbf{x}})$. Our goal is to fit a logistic regression working model for predicting *Y* with $(\varvec{X}^T,\varvec{Z}^T)^T$,

1 $$\begin{aligned} P_{\varvec{\beta }}(Y=1|\varvec{X}={\textbf{x}},\varvec{Z}={\textbf{z}})=g(\varvec{\beta }; {\textbf{x}},{\textbf{z}})\equiv \frac{\exp \left( \beta _0+\varvec{\beta }_{\varvec{X}}^T {\textbf{x}}+ \varvec{\beta }_{\varvec{Z}}^T {\textbf{z}}\right) }{1+\exp \left( \beta _0+\varvec{\beta }_{\varvec{X}}^T {\textbf{x}}+ \varvec{\beta }_{\varvec{Z}}^T {\textbf{z}}\right) }, \end{aligned}$$

where $\varvec{\beta }= (\beta _0, \varvec{\beta }_{\varvec{X}}^T, \varvec{\beta }_{\varvec{Z}}^T)^T$ are the unknown regression parameters, that calibrates well in $\mathcal {P}$. We assume that the conditional distribution of $\varvec{Z}$ given $\varvec{X}$ is the same in $\mathcal {P}$ and $\mathcal {P}_{\text {S}}$ and follows a parametric model $f_{\varvec{\tau }}({\textbf{z}}|{\textbf{x}})$. That is, ${\text {Pr}}\left( \varvec{Z}={\textbf{z}}\mid \varvec{X}={\textbf{x}}; \varvec{\tau }\right) ={\text {Pr}}_{S}\left( \varvec{Z}={\textbf{z}}\mid \varvec{X}={\textbf{x}}; \varvec{\tau }\right) =f_{\varvec{\tau }}({\textbf{z}}|{\textbf{x}})$, where $\varvec{\tau }$ is a vector of Euclidean parameters. Below we propose a constrained maximum likelihood method for fitting model (1) with data $\left( Y_{i}, \varvec{X}_{i}, \varvec{Z}_{i}\right)$, $i=1\ldots N$ from the study source population. The fitted model $P_{{\hat{\varvec{\beta }}}}(Y=1|\varvec{X}={\textbf{x}}, \varvec{Z}={\textbf{z}})$ is guaranteed to calibrate similarly well as model $\varphi (\varvec{X})$ in the target population $\mathcal {P}$ despite that the data is obtained from $\mathcal {P}_{\text {S}}$.

### Constrained maximum likelihood method (“cML”)

The log-likelihood function for the observed data $\left( Y_{i}, \varvec{X}_{i}, \varvec{Z}_{i}\right)$, $i=1\ldots N$, can be written as

2 $$\begin{aligned} \begin{aligned} l\left( \varvec{\theta }\right) \equiv \sum _{i=1}^N \log \left[ \frac{\exp \left\{ Y_{i} \left( \beta _0 +\varvec{\beta }_{\varvec{X}}^{T}\varvec{X}_{i} +\varvec{\beta }_{\varvec{Z}}^{T}\varvec{Z}_{i}\right) \right\} }{1 + \exp \left( \beta _0 + \varvec{\beta }_{\varvec{X}}^{T}\varvec{X}_{i} + \varvec{\beta }_{\varvec{Z}}^{T}\varvec{Z}_{i}\right) }{f_{\varvec{\tau }}}\left( \varvec{Z}_{i}\mid \varvec{X}_{i}\right) \right] , \end{aligned} \end{aligned}$$

where the marginal distribution of $\varvec{X}$ is ignored. The model fitted via direct maximization of likelihood function (2), denoted as $g({\hat{\varvec{\beta }}}^s; {\textbf{x}},{\textbf{z}})$, is expected to calibrate well in the source population $\mathcal {P}_{\text {S}}$ but not necessarily in the target population $\mathcal {P}$ if these two populations differ. The superscript “s” in $g({\hat{\varvec{\beta }}}^s; {\textbf{x}},{\textbf{z}})$ indicates that it was fitted solely using the source data. To address calibration in the target population $\mathcal {P}$, we propose that maximization of likelihood function (2) under the constraints that the predicted risk by $P_{{\hat{\varvec{\beta }}}}(Y=1|\varvec{X}={\textbf{x}}, \varvec{Z}={\textbf{z}})$ aligns closely with that by $\varphi (\varvec{X})$ within risk intervals defined by $\varvec{X}$. The constraint is formally constructed as follows. We first categorize the predicted risk by $\varphi (\varvec{X})$ into *I* intervals $(a_r, b_r)$, $r=1, \ldots I$, with $a_1=\min \{\varphi (\varvec{X}) \}$ and $b_I=\max \{\varphi (\varvec{X})\}$. Note that no specific functional form is required for $\varphi (\varvec{X})$. The averaged risk in $\mathcal {P}$ by $\varphi (\varvec{X})$ in $(a_r, b_r)$, $r=1, \ldots I$ can be written as

$$\begin{aligned} P^e_{r}\equiv \frac{\int _{a_{r}<\varphi \left( {\textbf{x}}\right) \le b_{r}}\varphi ({\textbf{x}})\delta \left( {\textbf{x}}\right) d{\textbf{x}}}{\int _{a_{r}<\varphi \left( {\textbf{x}}\right) \le b_{r}}\delta \left( {\textbf{x}}\right) d{\textbf{x}}},~~~~~r=1,...,I. \end{aligned}$$

The proposed constraints enforce that the difference between the averaged risk by the fitted model $g({\hat{\varvec{\beta }}}; {\textbf{x}},{\textbf{z}})$ and that by $\varphi (\varvec{X})$ be small in the target population. Let $\varvec{d} = \left( d_{1},\ldots ,d_{I}\right)$ denote a vector of positive numbers for the tolerance of difference. The constraints are formally expressed as

3 $$\begin{aligned} \left| \frac{\int _{a_{r}<\varphi \left( {\textbf{x}}\right) \le b_{r}}\int _{{\textbf{z}}^{\prime }}g(\varvec{\beta }; {\textbf{x}},{\textbf{z}}')\delta \left( {\textbf{x}}\right) f_{\varvec{\tau }}\left( {\textbf{z}}^{\prime }\mid {\textbf{x}}\right) d{\textbf{z}}^\prime d{\textbf{x}}}{\int _{a_{r}<\varphi \left( {\textbf{x}}\right) \le b_{r}}\delta \left( {\textbf{x}}\right) d{\textbf{x}}} - P_r^e\right| \le d_r\cdot P_r^e, \end{aligned}$$

$r=1,\ldots ,I$. Estimates of parameters $(\varvec{\beta }^T, \varvec{\tau }^T)^T$, denoted as $(\hat{\varvec{\beta }}^T, \hat{\varvec{\tau }}^T)^T$, can then be obtained by maximizing the likelihood function (2) under constraints (3).

The tolerance vector $\varvec{d}$ is pre-specified, where smaller values enforce stronger reliance on external information summarized in $P_r^e, r=1,\ldots , I$, in model fitting. A large value for $d_r$ can be specified to disable the constraint in the $r^{th}$ interval. Ideally, the selection of calibration intervals $(a_r, b_r)$ should align with that used for assessing calibration of $\varphi (\varvec{X})$, and they can be adjusted to allow for more relaxed or tighter constraints. For example, when the fitted model $g({\hat{\varvec{\beta }}}; {\textbf{x}},{\textbf{z}})$ is intended to be used for identifying high-risk patients, multiple calibration intervals can be placed in the high-risk region. The constraints (3) require that $P_r^e$ provide accurate estimates of the average risk in interval $(a_r, b_r)$. However, this may not always be the case. For example, BCRAT generally overestimates breast cancer risk in the high-risk region (Pal Choudhury et al. 2020). When the observed-to-expected ratio deviates from one in the validation of $\varphi (\varvec{X})$, $P_r^e$ can be adjusted by multiplying this ratio. This flexibility is particularly attractive given that mis-calibration frequently occurs in the low- or high-risk regions.

### Computation of the cML estimator

Our proposed procedures for obtaining cML estimates is summarized as follows:

1. Choose risk intervals $\{(a_r, b_r),\ r=1,\ldots I\}$ as defined by $\varphi (\varvec{X})$, and set the tolerance values $d_r$.
2. Obtain the distribution of conventional predictors $\varvec{X}$, $\delta \left( {\textbf{x}}\right)$, in the target population $\mathcal {P}$.
3. Maximize likelihood function (2) subject to the constraints (3)

In step 3, we apply the Lagrangian method based on Karush–Kuhn–Tucher (KKT) conditions (Deng et al. 2018; Nocedal and Wright 1999) to accommodate inequality in constraints (3). Let $\varvec{\theta }=\left( \varvec{\beta }^T,\varvec{\tau }^T\right) ^T$. Define two functions $C^+_{r}\left( \varvec{\theta }\right)$ and $C^{-}_{r}\left( \varvec{\theta }\right)$, $r=1,\ldots ,I$, as

$$\begin{aligned}&C^+_{r}\left( \varvec{\theta }\right) \\&\ \ \equiv \frac{\int _{a_{r}< \varphi \left( {\textbf{x}}\right) \le b_{r}}\int _{{\textbf{z}}^{\prime }} g(\varvec{\beta }; {\textbf{x}},{\textbf{z}}') \delta \left( {\textbf{x}}\right) f_{\varvec{\tau }}\left( {\textbf{z}}^{\prime }\mid {\textbf{x}}\right) d{\textbf{z}}^\prime d{\textbf{x}}}{\int _{a_{r}<\varphi \left( {\textbf{x}}\right) \le b_{r}}\delta \left( {\textbf{x}}\right) d{\textbf{x}}}-(1+d_r) P^e_{r},\\&C^-_{r}\left( \varvec{\theta }\right) \\&\ \ \equiv (1-d_r) P^e_{r} - \frac{\int _{a_{r}< \varphi \left( {\textbf{x}}\right) \le b_{r}}\int _{{\textbf{z}}^{\prime }} g(\varvec{\beta }; {\textbf{x}},{\textbf{z}}') \delta \left( {\textbf{x}}\right) f_{\varvec{\tau }}\left( {\textbf{z}}^{\prime }\mid {\textbf{x}}\right) d{\textbf{z}}^\prime d{\textbf{x}}}{\int _{a_{r}<\varphi \left( {\textbf{x}}\right) \le b_{r}}\delta \left( {\textbf{x}}\right) d{\textbf{x}}}. \end{aligned}$$

Let $\varvec{\Theta }\subseteq \mathbb {R}^{1+p+q+\left| \varvec{\tau }\right| }$ denote the parameter space for $\varvec{\theta }$ with $\left| \varvec{\tau }\right|$ being the length of $\varvec{\tau }$. We assume that $\varvec{\Theta }$ is bounded and connected. Let $\varvec{\Theta }_{\varvec{C}}$ denote the feasible region (Moore et al. 2008) that contains all points $\varvec{\theta }\in \varvec{\Theta }$ that satisfy constraints (3). $C^+_{r}$ and $C^-_{r}$ indicate the upper bound and lower bound of the inequality constraint (3), correspondingly. Then the cML estimates can be obtained as

4 $$\begin{aligned} \begin{aligned}&~~~\hat{\varvec{\theta }}=\text{ argmax}_{\varvec{\Theta }}~ l\left( \varvec{\theta }\right) \\ \text {subject to}&~~~C^+_{r}\left( \varvec{\theta }\right) \le 0,~~~C^-_{r}\left( \varvec{\theta }\right) \le 0,~~~ r=1,...,I. \end{aligned} \end{aligned}$$

### Asymptotic properties of ${\hat{\varvec{\theta }}}$ and $g({\hat{\varvec{\beta }}}; {\textbf{x}},{\textbf{z}})$

The proposed constraints (3) enforce requirements on the parameter space $\varvec{\Theta }$. We first study the asymptotic properties of $\varvec{\theta }$. Denote the true underlying model for $(Y, \varvec{X}, \varvec{Z})$ in ${\text {Pr}}_{S}$ as ${\text {Pr}}_{S} \left( Y=1 \mid \varvec{X}={\textbf{x}}, \varvec{Z}={\textbf{z}}\right) = h\left( {\textbf{x}}, {\textbf{z}}\right)$, ${\text {Pr}}_{S} \left( \varvec{Z}= {\textbf{z}}\mid \varvec{X}={\textbf{x}}\right) =f_{\varvec{\tau }_{0}}\left( {\textbf{z}}\mid {\textbf{x}}\right)$, and ${\text {Pr}}_{S}\left( X = {\textbf{x}}\right) = \pi _{0}\left( {\textbf{x}}\right)$, where the function $h\left( {\textbf{x}},{\textbf{z}}\right)$ is unspecified and allowed to differ from the working model (1). The true conditional density function of $\left( Y, Z\right)$ given $\varvec{X}$ can be written as

5 $$\begin{aligned} {\text {Pr}}_{S}\left( Y=y,\varvec{Z}={\textbf{z}}\mid \varvec{X}={\textbf{x}}\right) =h\left( {\textbf{x}},{\textbf{z}}\right) ^{y}\left\{ 1-h\left( {\textbf{x}},{\textbf{z}}\right) \right\} ^{1-y}f_{\varvec{\tau }_{0}}\left( {\textbf{z}}\mid {\textbf{x}}\right) . \end{aligned}$$

cML does not require an explicit model for $(Y|\varvec{X}, \varvec{Z})$ in the target population $\mathcal {P}$, and is obtained by maximizing the following working likelihood function

$$\begin{aligned} p_{\varvec{\theta }}\left( y,{\textbf{z}}\mid {\textbf{x}}\right) =g(\varvec{\beta };{\textbf{x}}, {\textbf{z}})^{y}\left\{ 1-g(\varvec{\beta }; {\textbf{x}},{\textbf{z}})\right\} ^{1-y} f_{\varvec{\tau }}\left( {\textbf{z}}\mid {\textbf{x}}\right) , \end{aligned}$$

subject to constraints (3). Assume the standard regularity condition that $E_{S}\left[ \log \left\{ {\text {Pr}}_{S}\left( Y, \varvec{Z}\mid \varvec{X}\right) \right\} \right]$ and $E_{S}\left[ \log \left\{ p_{\varvec{\theta }}\left( Y, \varvec{Z}\mid \varvec{X}\right) \right\} \right]$ exist for all $\varvec{\theta }\in \varvec{\Theta }$, and define the Kullback-Liebler Information Criterion (KLIC) as

6 $$\begin{aligned} I\left( {\text {Pr}}_{S}:p_{\varvec{\theta }}\right) =: E_{S}\left[ \log \left\{ {\text {Pr}}_{S}\left( Y,\varvec{Z}\mid \varvec{X}\right) /p_{\varvec{\theta }}\left( Y,\varvec{Z}\mid \varvec{X}\right) \right\} \right] . \end{aligned}$$

The consistency of ${\hat{\varvec{\theta }}}$ is established as follows.

#### Theorem 1

Assume that $\varvec{\Theta }$ is connected and bounded and that $I \left( {\text {Pr}}_{S}:p_{\varvec{\theta }}\right)$ has a unique minimum at $\varvec{\theta }^{*}\in \varvec{\Theta }_{C}$. The cML estimator $\hat{\varvec{\theta }}$ is consistent, $\hat{\varvec{\theta }} {\mathop {\rightarrow }\limits ^{p}}\varvec{\theta }^*$.

The inequality constraint $C_{r}^{+}\left( \varvec{\theta }\right) \le 0$ or $C_{r}^{-}\left( \varvec{\theta }\right) \le 0$ is active at a feasible point $\varvec{\theta }\in \varvec{\Theta }_{\varvec{C}}$ only if $C_{r}^{+}\left( \varvec{\theta }\right) = 0$ or $C_{r}^{-}\left( \varvec{\theta }\right) = 0$. Let $\textbf{C}_{\oplus }\left( \varvec{\theta }\right)$ represent the active constraints at $\varvec{\theta }$. That is, the vector $\textbf{C}_{\oplus }\left( \varvec{\theta }\right)$ consists of $\{C_{i}^{+}\left( \varvec{\theta }\right) , i\in K^{+} \left( \varvec{\theta }\right) \} \cup \{C_{j}^{-}\left( \varvec{\theta }\right) , j\in K^{-} \left( \varvec{\theta }\right) \}$, where $K^{+} \left( \varvec{\theta }\right) ,~K^{-}\left( \varvec{\theta }\right) \subset \{ 1,\ldots ,I\}$, $K^{+}\left( \varvec{\theta }\right) \cap K^{-}\left( \varvec{\theta }\right) =\emptyset$, $C_{i}^+\left( \varvec{\theta }\right) =0$ if $i\in K^{+}\left( \varvec{\theta }\right)$, $C_{i}^+\left( \varvec{\theta }\right) <0$ otherwise, and $C_{j}^-\left( \varvec{\theta }\right) =0$ if $j\in K^{-}\left( \varvec{\theta }\right)$, $C_{j}^-\left( \varvec{\theta }\right) <0$, otherwise. Define $\Xi \left( \varvec{\theta }\right)$ as a matrix whose columns form an orthonormal basis for the null space of $\partial \textbf{C}_{\oplus }\left( \varvec{\theta }\right) /\partial \varvec{\theta }$, namely,

$$\begin{aligned} \Xi \left( \varvec{\theta }\right) ^{T}\partial \textbf{C}_{\oplus }\left( \varvec{\theta }\right) ^{T}/\partial \varvec{\theta }=\varvec{0},~~\textrm{and}~~\Xi \left( \varvec{\theta }\right) ^{T}\Xi \left( \varvec{\theta }\right) =\varvec{I}. \end{aligned}$$

Assume that $\varvec{\theta }^*$ is a regular point of the active constraints, that is, the gradient matrix of $\partial \textbf{C}_{\oplus } \left( \varvec{\theta }^*\right) ^{T}/\partial \varvec{\theta }$ has full row rank $\left| K^{+}\left( \varvec{\theta }^*\right) \right| +\left| K^{-}\left( \varvec{\theta }^{*}\right) \right|$. Define $\mathcal {I} \left( \varvec{\theta }\right) := E_S\left[ \{\partial l_1 \left( \varvec{\theta }\right) /\partial \varvec{\theta }\}^{\otimes 2}\right]$, where $l_1 \left( \varvec{\theta }\right)$ denotes the first summand in $l\left( \varvec{\theta }\right)$, namely, $l_1\left( \varvec{\theta }\right) =\log \left\{ p_{\varvec{\theta }}\left( Y_1,\varvec{Z}_1\mid \varvec{X}_1\right) \right\} .$ We derive the large sample distribution of ${\hat{\varvec{\theta }}}$ and show the consistency of individual risk estimates in Theorem 2.

#### Theorem 2

Assume the same regularity conditions as Theorem 1, and further assume that $\Xi \left( \varvec{\theta }^*\right) ^{T} E_S\{\frac{\partial ^2 l_1\left( \varvec{\theta }^*\right) }{\partial \varvec{\theta }\partial \varvec{\theta }^T}\}\Xi \left( \varvec{\theta }^*\right)$ is nonsingular. We can show that ${\hat{\varvec{\theta }}}$ is asymptotically normally distributed,

$$\begin{aligned} \sqrt{N}\left( \hat{\varvec{\theta }}-\varvec{\theta }^*\right) {\mathop {\rightarrow }\limits ^{D}} \textbf{V}\left( \varvec{\theta }^*\right) \mathcal {I}\left( \varvec{\theta }^*\right) \textbf{V}\left( \varvec{\theta }^*\right) ^{T}, \end{aligned}$$

where $\textbf{V}\left( \varvec{\theta }^*\right)$ is expressed as

$$\begin{aligned} \textbf{V}\left( \varvec{\theta }^*\right) =\Xi \left( \varvec{\theta }^*\right) \left[ \Xi \left( \varvec{\theta }^*\right) ^{T}E_S \left\{ \frac{\partial ^2 l_1\left( \varvec{\theta }^*\right) }{\partial \varvec{\theta }\partial \varvec{\theta }^T} \right\} \Xi \left( \varvec{\theta }^*\right) \right] ^{-1}\Xi \left( \varvec{\theta }^*\right) ^{T}. \end{aligned}$$

#### Corollary 1

(Consistency of individual risk estimates) We make the same assumptions as Theorems 1 and 2. Further we assume that the link function *g* satisfies $\inf \limits _{x\in \mathbb {R}}g^{\prime }\left( x\right) \ge 0$, $\sup \limits _{x\in \mathbb {R}}g^{\prime }\left( x\right) <M$, and $\sup \limits _{x\in \mathbb {R}}g^{\prime \prime }\left( x\right) <M$, where *M* is a positive constant. Suppose $\varvec{u}_{\text {new}} = \left( {\textbf{x}}_{\text {new}}^{T}, {\textbf{z}}_{\text {new}}^{T} \right) ^{T}$ is sub-Gaussian random vector satisfying $\sup \limits _{\left\Vert \varvec{v}\right\Vert =1}\varvec{v}^{T}E\left( \varvec{u}_{\text {new}} \varvec{u}_{\text {new}}^{T}\right) \varvec{v}\le \sigma _{\text {max}}$ where $\sigma _{\text {max}}$ is a positive constant. The following result holds,

7 $$\begin{aligned} g\left( \varvec{u}_{\text {new}}^{T}\widehat{\varvec{\beta }}\right) -g\left( \varvec{u}_{\text {new}}^{T}\varvec{\beta }^{*}\right) =O_{p}\left( \left\Vert \widehat{\varvec{\beta }}-\varvec{\beta }^{*}\right\Vert \right) =o_{p}\left( 1\right) . \end{aligned}$$

### Further considerations on $\varvec{\theta }^*$

We consider two special cases to provide insights on $\varvec{\theta }^*$. In the first case, the target population $\mathcal {P}$ is identical to the source population $\mathcal {P}{_{\text {S}}}$, and the model (1) is correctly specified. Then $\varvec{\theta }^*$ is the true parameter value in the target population. cML is expected to be more efficient than the unconstrained ML estimator. This result aligns with the literature on integrating external summary data to increase statistical efficiency (Chatterjee et al. 2016; Zheng et al. 2022a, b; Zhai and Han 2022) mentioned earlier. In the second case, the target population $\mathcal {P}$ is different from the study source population $\mathcal {P}_S$, but model (1) holds in both $\mathcal {P}$ and $\mathcal {P}_S$ with different parameter values $\varvec{\theta }_{S}$ and $\varvec{\theta }_{T}$. If $\mathcal {P}_{S}$ is not too far away $\mathcal {P}$, namely, $\left\Vert \varvec{\theta }_{T}-\varvec{\theta }_{S}\right\Vert <\eta$ for some small $\eta >0$, we can show that cML parameter $\varvec{\theta }^*$ can be approximately obtained as follows. Let $p_{\varvec{\theta }_{T}}$ denote Pr$\left( Y=y,\varvec{Z}={\textbf{z}}\mid \varvec{X}={\textbf{x}}\right)$, $p_{\varvec{\theta }_S}$ denote ${\text {Pr}}_S\left( Y=y,\varvec{Z}={\textbf{z}}\mid \varvec{X}={\textbf{x}}\right)$, and $g_{1}\left( \varvec{\theta }; {\textbf{x}},{\textbf{z}}\right)$ denote $g(\varvec{\beta }; {\textbf{x}},{\textbf{z}}) f_{\varvec{\tau }}\left( {\textbf{x}}\mid {\textbf{z}}\right)$. Then by applying Taylor’s series expansion, and assuming that $\varphi \left( \varvec{X}\right)$ is very close to ${\text {Pr}}\left( Y=1\mid \varvec{X}\right)$, we can show that the cML parameter $\varvec{\theta }^*$ can be approximately obtained by maximizing the following approximate objective function

8 $$\begin{aligned}&\left( \varvec{\theta }-\varvec{\theta }_{S}\right) ^TE_S\left[ \frac{\partial \log \left\{ p_{\varvec{\theta }_{S}}\left( Y,Z\mid \varvec{X}\right) \right\} }{\partial \varvec{\theta }}\right] \nonumber \\&\qquad +\left( \varvec{\theta }-\varvec{\theta }_{S}\right) ^TE_S\left[ \frac{\partial ^2\log \left\{ p_{\varvec{\theta }_{S}}\left( Y,Z\mid \varvec{X}\right) \right\} }{\partial \varvec{\theta }\partial \varvec{\theta }^T}\right] \left( \varvec{\theta }-\varvec{\theta }_{S}\right) \end{aligned}$$

subject to constraints

$$\begin{aligned} \left| \frac{\left( \varvec{\theta }-\varvec{\theta }_{T}\right) ^T\int _{a_r<\varphi \left( {\textbf{x}}\right) \le b_r} \left\{ \partial g_{1}\left( \varvec{\theta }_{T}; {\textbf{x}},{\textbf{z}}\right) /\partial \varvec{\theta }\right\} \delta \left( {\textbf{x}}\right) d{\textbf{z}}d{\textbf{x}}}{\int _{a_r<\varphi \left( {\textbf{x}}\right) \le b_r}\delta \left( {\textbf{x}}\right) d{\textbf{x}}}\right| \le d_rP_r^e, ~~r=1,\ldots , I. \end{aligned}$$

**Fig. 1:**
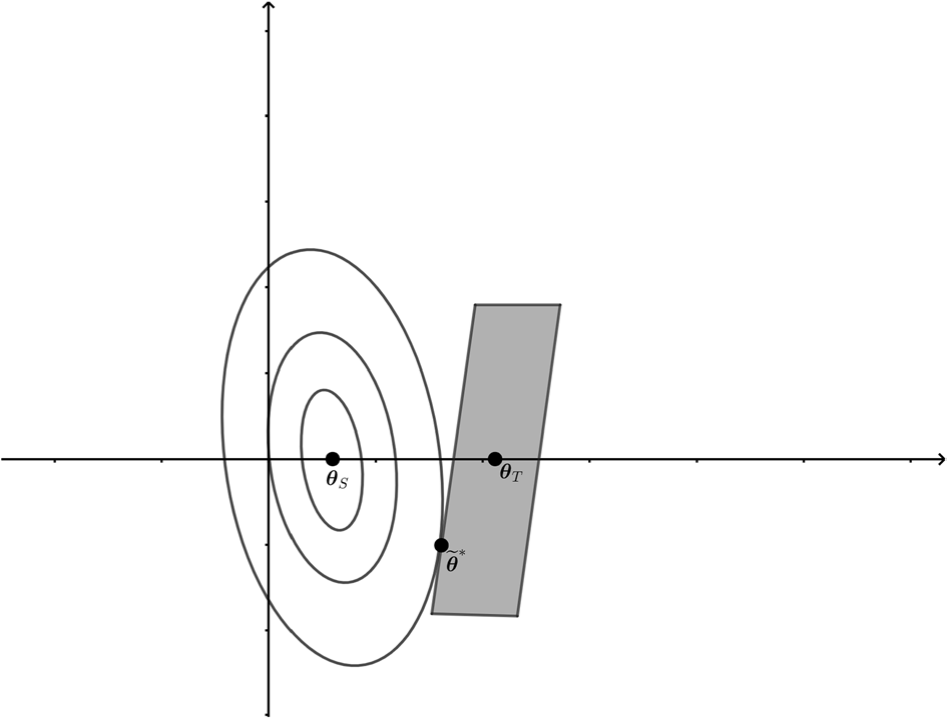
The elliptical contours of the objective functon (8) are shown by full curves, which are centered at $\varvec{\theta }_{S}$. The constrained region is the quadrangle centered at $\varvec{\theta }_{T}$. ${\tilde{\varvec{\theta }}}^*$ is the first position that the contours touch the quadrangle

Denote ${\tilde{\varvec{\theta }}}^{*}$ be the corresponding solution. It is reasonable to expect that under suitable regularity conditions, ${\tilde{\varvec{\theta }}}^{*}$ approximately converges to $\varvec{\theta }^*$. To get further insight into $\varvec{\theta }^*$, we draw the elliptical contours of the function (8) by the full curves in Fig. 1, which are centered at $\varvec{\theta }_{S}$. The constrained region is the quadrangle centered at $\varvec{\theta }_{T}$. ${\tilde{\varvec{\theta }}}^{*}$ is the first point that the contours touch the quadrangle.

An interesting question arises from Fig. 1. If $\varvec{\theta }_{S} = \left( \beta _{S0},\varvec{\beta }_{S\varvec{X}}^T, \varvec{\beta }_{S\varvec{Z}}^T, \varvec{\tau }_{S}^T\right) ^T$ and $\varvec{\theta }_{T} = \left( \beta _{T0}, \varvec{\beta }_{T\varvec{X}}^T, \varvec{\beta }_{T\varvec{Z}}^T, \varvec{\tau }_{T}^T\right) ^T$ only differ in the first coordinate, and consider $\beta _{S0}< \beta _{T0}$ without loss of generality. One may hope that $\varvec{\theta }^*$ would lie between $\varvec{\theta }_S$ and $\varvec{\theta }_T$, that is, $\varvec{\theta }^*=\left( \beta _{0}^*,\varvec{\beta }_{\varvec{X}}^*,\varvec{\beta }_{Z}^*,\varvec{\tau }^*\right)$ can satisfy conditions $\beta _{S0} \le \beta _{0}^* \le \beta _{T0}$ and $\left( \varvec{\beta }_{\varvec{X}}^{*T},\varvec{\beta }_{Z}^{*T},\varvec{\tau }^{*T}\right) ^T=\left( \varvec{\beta }_{S\varvec{X}}^T,\varvec{\beta }_{S\varvec{Z}}^T,\varvec{\tau }_{S}^T\right) ^T$. But Fig. 1 indicates that this is not necessarily the case.

## Breast cancer risk prediction using data from Penn Medicine Biobank

We applied the proposed method to analyze data from Penn Medicine Biobank (McCarthy et al. 2021) to assess the added value of breast density (“BD”), as measured by percent mammographic density, for predicting the 5-year risk of breast cancer following a negative screening mammogram. The study cohort consisted of 11, 370 Caucasian women in the age range of $40 \sim 84$ years who did not have prior history of breast cancer but underwent screening mammography in the University of Pennsylvania health system between years 2006 and 2015. Women who developed invasive breast cancer within 5 years of the screening mammogram were considered cases ($n=209$). We used the same numerical coding as in BCRAT for conventional predictors, including age at first live birth (“Ageflb"), age at menarche (“Agemen”), number of previous breast biopsies (“Nbiops”) and number of first-degree relatives (“Numrel”) who had breast cancer. Compared to women in the National Health Interview Survey (NHIS) who are representative of the general US female population, the women in the Penn Biobank tended to have a stronger family history of breast cancer, underwent more frequent biopsy examinations, and had their first live child at an older age (Table 1). Because BCRAT has been extensively validated (Bondy et al. 1994; Costantino et al. 1999; Rockhill et al. 2001) for estimating the absolute breast cancer risk within a specified age period, we used it to derive a base model. Subsequently, we applied the proposed method to Penn Biobank data for Caucasian women to develop a model that use both conventional predictors and BD to predict 5-year risk. This newly developed model is expected to calibrate similarly as BCRAT in the U.S. woman population.

Let $(T_1, T_2)$ denote the 5-year age interval with $T_2-T_1=5$ and $T_1$ and $T_2$ being integers, and $\varvec{X}$ denote Ageflb, Agemen, Nbiops, and Numrel. We constructed the constraints based on 5-year absolute risks of breast cancer estimated from BCRAT on the website https://dceg.cancer.gov/tools/risk-assessment/bcra (Gail et al. 1989), denoted as ${\tilde{\varphi }}(T_1, T_2; \varvec{X})$. We calculated $\varphi (\varvec{X})$ as the weighted average of the estimated risk for all 5-year intervals with $T_1 \in (25, 70)$, $\sum _{T_1 \in (25, 70)} {\tilde{\varphi }}(T_1, T_2;\varvec{X}) {\text {Pr}}([T_1,T_2])$ where ${\text {Pr}}([T_1,T_2])$, the proportion of women in age interval $[T_1, T_2]$, was estimated from NHIS. We chose quartiles of $\varphi (\varvec{X})$ as the endpoints of four constraint intervals (3) and calculated $P_r^e, r=1,2,3,4$. Moreover, the distribution of $\varvec{X}$ was estimated from NHIS and was treated as fixed quantities in the current analyses. The averaged 5-year risk within each of the four risk intervals is obtained as

$$\begin{aligned} \{{\widehat{P}}_r^e,r=1,\ldots ,4\}&=\{\widehat{{\text {Pr}}}(Y=1|\varphi (\varvec{X}) \in (a_r,b_r]), r=1,\ldots ,4\}\\&=\{1.1\%, 2.1\%, 3.4\%, 5.3\%\}, \end{aligned}$$

where we set $d_r=0.1, \ r=1,\ldots , 4$ so that the tolerance thresholds equaled $\{0.1*\widehat{P^e_r}, r=1,\ldots , 4\}$. Let *A* denote age. We assumed that the distribution of BD (“*Z*”), $f_{\varvec{\tau }}(\varvec{Z}={\textbf{z}}|\varvec{X}={\textbf{x}}, A=a)$, was the same in the Penn Biobank and U.S. woman population. In our analysis, based on the observations of breast density *Z* in (0, 1), we used the truncated log-normal distribution with constant variance for BD, where $\log Z \sim N(u,\sigma ^2)$ truncated on $(-\infty ,0)$, with $u = (\varvec{\tau }_{\text {mean}})^T (1,\varvec{X}^T, A)^T$. Here $\varvec{\tau }=(\varvec{\tau }_{\text {mean}}^T,\sigma )^T$. In the logistic regression model (1), we included an ordinal variable $Z^c$ instead of *Z*, which was created by assigning integer values $0\sim 9$ to the 10 intervals of *Z*, $(0,0.1], (0.1,0.2], \ldots , (0.8,0.9], (0.9,1)$, respectively.

We conducted four sets of analyses to fit model (1). The first is standard logistic regression analyses with both $\varvec{X}$ and *Z* that included all 11, 370 women, referred to as “Standard” Model. The maximum likelihood analysis was applied to fit model $f({\textbf{z}}|{\textbf{x}},a)$, which was needed for model evaluation. The expected number of cases based on the Standard model was calculated as

$$\begin{aligned} \frac{\sum _{{\textbf{x}}:a_{r}<\varphi \left( {\textbf{x}}\right) \le b_{r}} \sum _{{\textbf{z}}} \sum _{a}P_{\hat{\varvec{\beta }}}(Y=1|\varvec{X}={\textbf{x}},\varvec{Z}={\textbf{z}}) \delta _{{\textbf{x}}}f_{\hat{\varvec{\tau }}} \left( {\textbf{z}}\mid {\textbf{x}}, a \right) {\text {Pr}}(A=a)}{\sum _{{\textbf{x}}: a_{r}<\varphi \left( {\textbf{x}}\right) \le b_{r}} \delta _{{\textbf{x}}}}. \end{aligned}$$

The averaged risks by the Standard Model and those in the U.S. Caucasian woman population differed by 16% and 29% in the two high-risk calibration intervals, indicating lack of calibration of the Standard Model in the U.S. Caucasian woman population. This discrepancy can be partially explained by the difference in the distribution of predictors between Penn Biobank data and the NHIS as shown in Table 1, primarily in the distribution of Nbiops ($X_3$) and Numrel ($X_4$). Next, we applied cML considering two sets of constraints, the quartiles or $(50\%, 70\%, 90\%)$ percentiles of $\varphi (\varvec{X})$. Note that the latter imposed finer constraints in the tail of the risk distribution to ensure improved calibration in the high risk region. Lastly, to explore the performance of the proposed method when the source data is further away from the target population, we repeated the above analyses in a subset of the data that contained all cases and 90% of the women whose BCRAT risk was above $1.67\%$.

The results are presented in Table 2. The log odds ratio parameter estimates obtained by the proposed method using quartiles (“$\hbox {cML}^1$”) or the 50%, 70%, and 90% percentiles (“$\hbox {cML}^2$”) of $\varphi (\varvec{X})$ as constraints can be quite different from those by the standard methods (“Standard”). For example, the Standard log odds ratio parameter estimate for Nbiops was 0.462 but became 0.257 by $\hbox {cML}^1$. The difference between $\hbox {cML}^1$ and $\hbox {cML}^2$ was somewhat larger with the full data than with the subsample. Interestingly, both $\hbox {cML}^1$ and $\hbox {cML}^2$ were similar in the analyses with the full data or subsample, even when the Standard estimates differed substantially. The parameter estimates for BD and those in the truncated log-normal distribution were similar, although there was minor difference when comparing the full data and subsample results. By the Wald test, Nbiops ($X_3$) and Numrel ($X_4$) were significant in all analyses, Ageflb ($X_1$) was significant by $\hbox {cML}^1$ in the full data analysis and by $\hbox {cML}^2$ in the subsample analysis. BD was not significant in any analyses.

**Fig. 2:**
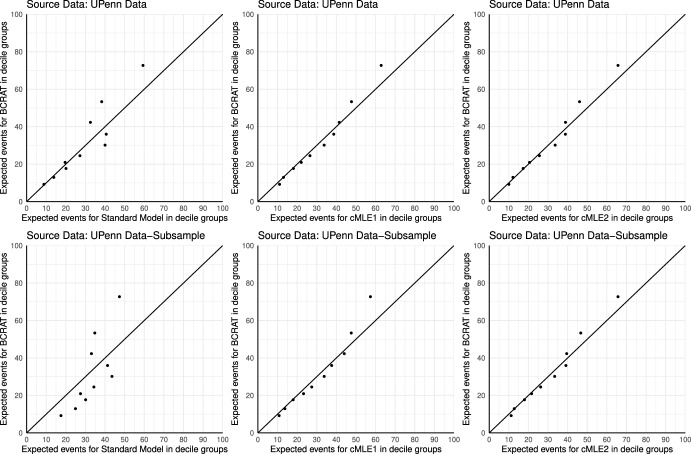
Calibration plots for models fitted using Standard, $\hbox {cML}^1$ and $\hbox {cML}^2$ methods, with BCRAT as the Benchmark

To show that the fitted model by the proposed method indeed led to improved calibration, we computed the expected number of cases per 100, 000 women based on risk estimates from different models in various woman subgroups. We used the expected numbers from $\varphi (\varvec{X})$ as benchmark. In the analysis of the selected subset (“Subsample”), at the benchmark of 1, 263, the expected number was 2, 212 from the Standard Model, which decreased to 1, 396 by $\hbox {cML}^1$ and 1, 370 by $\hbox {cML}^2$. Therefore, although all three models over-predicted the number of cases, $\hbox {cML}^1$ and $\hbox {cML}^2$ were very close to the benchmark. Figure 2 displays calibration plots indicating the expected events vs observed events of invasive breast cancer cases in each decile of risk. In the absence of independent validation data, the expected number of events for $\varphi (\varvec{X})$ were used as the “observed” number. The Standard model appeared to be ill-calibrated, particularly in the high risk region. The models fitted using the proposed method achieved improved calibration. When the analyses were repeated in the subsample, model $\hbox {cML}^1$ under-predicted the number of cases in the high risk interval, whereas $\hbox {cML}^2$ showed much improved calibration due to finer constraints in this region (Table 3).

**Table 1:** Estimated marginal distributions of predictors in Penn Biobank and NHIS

		Penn Biobank (%)	NHIS (%)
Age at screening (*A*)	< 40	0	10
	40–49	22.4	28.6
	$\geqslant$ 50	77.6	61.4
Ageflb ($X_1$)	< 20	3.9	19.8
	20–24	16.1	35.6
	25–29 or nulliparous	56.5	34.0
	$\geqslant$ 30	23.5	10.6
Agemen ($X_2$)	$\geqslant$14	23.2	28.3
	12–13	58.5	55.1
	< 12	18.3	16.6
Nbiops ($X_3$)	0	70.6	85.3
	1	21.4	10.7
	$\geqslant$ 2	8.0	4.0
Numrel ($X_4$)	0	78.2	88.2
	1	19.6	10.8
	$\geqslant$ 2	2.2	1.0

**Table 2:** Estimated log odds ratio parameters

	Penn (*N*=11,370)			Penn subsample (*N*=7,089)		
	Standard model	$\hbox {cMLE}^1$(SD)	$\hbox {cMLE}^2$(SD)	Standard model	$\hbox {cMLE}^1$(SD)	$\hbox {cMLE}^2$(SD)
$\beta _{0}$	− 5.162 (0.271)	− 4.733 (0.222)	− 4.863 (0.128)	− 4.389 (0.271)	− 4.805 (0.135)	− 4.717 (0.156)
$\beta _{X_1}$	0.264 (0.101)	0.071 (0.072)	0.066 (0.010)	0.210 (0.100)	0.134 (0.015)	0.024 (0.032)
$\beta _{X_2}$	0.143 (0.110)	0.110 (0.114)	0.153 (0.117)	0.127 (0.111)	0.139 (0.134)	0.170 (0.139)
$\beta _{X_3}$	0.462 (0.094)	0.257 (0.071)	0.334 (0.009)	0.297 (0.093)	0.171 (0.010)	0.318 (0.087)
$\beta _{X_4}$	0.563 (0.117)	0.657 (0.074)	0.659 (0.004)	0.234 (0.121)	0.697 (0.004)	0.621 (0.085)
$\beta _{Z^c}$	0.081 (0.058)	0.079 (0.060)	0.078 (0.062)	0.073 (0.059)	0.061 (0.071)	0.061 (0.071)
$\tau _{\text {mean}\_0}$	− 1.878 (0.023)	− 1.879 (0.023)	− 1.879 (0.023)	− 1.868 (0.030)	− 1.869 (0.030)	− 1.868 (0.030)
$\tau _{\text {mean}\_{X_1}}$	0.081 (0.008)	0.081 (0.008)	0.081 (0.008)	0.076 (0.010)	0.076 (0.010)	0.076 (0.010)
$\tau _{\text {mean}\_{X_2}}$	− 0.098 (0.009)	− 0.098 (0.009)	− 0.098 (0.009)	− 0.085 (0.011)	− 0.085 (0.011)	− 0.085 (0.011)
$\tau _{\text {mean}\_{X_3}}$	0.074 (0.009)	0.075 (0.009)	0.074 (0.009)	0.065 (0.010)	0.065 (0.010)	0.065 (0.010)
$\tau _{\text {mean}\_{X_4}}$	0.033 (0.012)	0.034 (0.012)	0.034 (0.012)	0.032 (0.013)	0.032 (0.013)	0.032 (0.013)
$\tau _{\text {mean}\_{A}}$	− 0.066 (0.003)	− 0.066 (0.003)	− 0.066 (0.003)	− 0.065 (0.004)	− 0.065 (0.004)	− 0.065 (0.004)
$\sigma$	0.593 (0.004)	0.593 (0.004)	0.593 (0.004)	0.595 (0.005)	0.595 (0.005)	0.595 (0.005)

“Standard”: estimates obtained by maximizing log-likelihood (2); $\hbox {cML}^1$: estimates by the proposed method using quartiles of 5-year BCRAT risk $\varphi ({\textbf{x}})$ to define the constraints; $\hbox {cML}^2$: the same as $\hbox {cML}^1$ but using $(50\%, 70\%, 90\%)$ percentiles of $\varphi ({\textbf{x}})$ to define the constraints

**Table 3:** Expected number of breast cancer cases per 100, 000 women based on predictions from BCRAT, “Standard”, $\hbox {cML}^1$ and $\hbox {cML}^2$

	Full Data (*N*=11,370)				Subsample (*N*=7,089)			
	BCRAT	Standard	$\hbox {cML}^1$	$\hbox {cML}^2$	BCRAT	Standard	$\hbox {cML}^1$	$\hbox {cML}^2$
All women	1263	1285	1378	1277	1263	2212	1396	1370
Nbiops								
0	1187	1141	1293	1173	1187	2072	1340	1265
1	1522	1825	1706	1670	1522	2783	1617	1769
$\geqslant$ 2	2190	2908	2317	2439	2190	3671	2005	2531
Numrel								
0	1108	1160	1220	1128	1108	2134	1226	1222
1	2204	2076	2365	2201	2204	2732	2459	2289
$\geqslant$ 2	4709	3779	4622	4375	4709	3507	4843	4377

## Simulation studies

We conducted extensive simulation studies to evaluate the finite sample performance of the proposed methods. We first define a target population through the distribution of $(Y, \varvec{X}, Z)$, which was then distorted for use to generate study data for model development. The data generation scheme for $\varvec{X}$ is summarized in Table 4. Parameters for the target population were chosen to be similar to those observed in the NHIS. We generated *Z* from the log-normal distribution $\log Z \sim N(\mu , \sigma ^2)$ truncated on $(-\infty , 0)$, where $\mu =(\varvec{\tau }_{\text {mean}})^T (1,\varvec{X}^T)^T$ with $\varvec{\tau }_{\text {mean}} = (-2, 0.1, -0.1, 0.1, 0.1)$ and $\sigma = 0.6$. Here $\varvec{\tau }=(\varvec{\tau }_{\text {mean}}^T,\sigma )^T$. A categorized version of *Z*, denoted as $Z^c$, was created to take integer values $0\sim 9$ corresponding to the 10 intervals $(0.1t, 0.1(t+1)]$, $t=0, \ldots , 9$. Data for the outcome variable *Y* was generated from model (1) using predictors $\varvec{X}$ and $Z^c$, with the corresponding parameter values set at $(-0.5, 0.4, 0.3, 0.65, 0.1)$. The intercept parameter $\beta _0$ was chosen to achieve the outcome prevalence ${\text {Pr}}(Y=1) = 0.1$.

**Table 4:** The distribution of $\varvec{X}$ in the simulated target and source populations

	Generation scheme in the target population	Generation scheme in the source population	
	Scenario I/II/III	Scenario I	Scenario II/III
$X_1$	Multinomial distribution with probabilities (0.2, 0.36, 0.34, 0.1)	Multinomial distribution with probabilities (0.2, 0.36, 0.34, 0.1)	Multinomial distribution with probabilities (0.05, 0.16, 0.56, 0.23)
$X_2$	Multinomial distribution with probabilities (0.28, 0.55, 0.17)	Multinomial distribution with probabilities (0.28, 0.55, 0.17)	Multinomial distribution with probabilities (0.23, 0.59, 0.18)
$X_3$	Taking values 0, 1, 2 corresponding to values 0, 1, or $\geqslant$ 2 generate from Poisson(0.3) distribution	Taking values 0, 1, 2 corresponding to values 0, 1, or $\geqslant$ 2 generate from Poisson(0.3) distribution	Taking values 0, 1, 2 corresponding to values 0, 1, or $\geqslant$ 2 generate from Poisson(0.5) distribution
$X_4$	Taking values 0, 1, 2 corresponding to values 0, 1, or $\geqslant$ 2 generate from Poisson(0.2) distribution	Taking values 0, 1, 2 corresponding to values 0, 1, or $\geqslant$ 2 generate from Poisson(0.2) distribution	Taking values 0, 1, 2 corresponding to values 0, 1, or $\geqslant$ 2 generate from Poisson(0.2) distribution

We generated a large dataset to fit a logistic regression model for *Y* given $\varvec{X}$ to use as $\varphi (\varvec{X})$. We chose the quartiles of $\varphi (\varvec{X})$ to set the constraints when applying cML. The data generating distribution of $\varvec{X}$ in the target population, $\delta \left( {\textbf{x}}\right)$, was assumed known in the analyses. The calibration benchmark $P_r^e$, $r=1,\ldots 4$, was calculated as $\sum _{i=1}^{m} I\{y_i=1,a_{r} \le \varphi ({\textbf{x}}_i) \le b_{r}\}/\sum _{i=1}^{m} I\{a_{r} \le \varphi ({\textbf{x}}_i) \le b_{r}\}$ from a cross-sectional sample of size $m=50,000$ that was randomly drawn from the target population. The tolerance threshold in (3) was set at $d=0.1$.

We generated three sets of study data of size $N=2000$. “Scenario I” data was generated from the same distribution as the target population. This scenario was designed to assess the performance of cML when the target and source populations are identical. For “Scenario II” data, $(\varvec{X}, Z)$ was generated using different parameter values, and *Y* was generated from the same model except that the intercept parameter was adjusted so that the prevalence of *Y* in the source population, ${\text {Pr}}_{{\text {S}}}(Y=1)$, was 1.5 times higher than that in the target population. This scenario was designed to mimic a setting where the source data was a biased sample. “Scenario III” data was generated using the same predictor distribution as Scenario II, but *Y* was generated from a different model for *Y* that was re-calibrated from that for Scenario II. The model takes the form $\text {logit}\{{\text {Pr}}_{{\text {S}}} (Y=1|{\textbf{x}},z)\} = a + b(\alpha +\varvec{\beta }_X^T {\textbf{x}}+ \beta _Z^T {\textbf{z}})$, where $\alpha$ was the intercept parameter used for generating the Scenario II data. We considered different values for (*a*, *b*), $a \ne 0$ and $b \ne 1$. For each dataset, we conducted three sets of analyses, the “Standard” method that directly maximizes the likelihood function (2) with truncated log-normal distribution for *Z*, and the cML method as specified above. We repeated the simulation 1, 000 times.

Results on the estimation of regression coefficients are summarized in Tables 5 and 6. Comparison between Standard and cML estimates can help reveal the effect of constraints on model fitting. With Scenario I data, both cML and Standard estimates for $\varvec{X}$ were close to the true parameter values, with cML having slightly smaller variance. cML can have larger efficiency gain when the tolerance threshold in the constraint is lower (data unreported). With Scenario II data, all cML estimates for $\varvec{X}$ are more or less away from the true values, while Standard estimates are nearly identical to the true values except for the intercept parameter as expected. Notably, the estimate of the intercept parameter by cML is $-2.44$ which is close to the true value $-2.40$. With Scenario III data, the cML estimates for $\varvec{X}$ became further away from the true values. The Standard estimates all differed from the truth as well. Interestingly, with $a=-0.5$ and $b=1.2$, the difference between the cML and true values became much larger and in the opposite direction compared to that for Standard estimates. In all scenarios, the averaged cML and Standard estimates were similar for $\beta _{Z^c}$, and for parameters $\varvec{\tau }=(\varvec{\tau }_{\text {mean}}^T, \sigma )^T$ in the model for *Z*. In all simulation scenarios, the averaged standard error (“ASE”) estimates were close to the empirical standard errors (“SE”) for cML in all three scenarios.

**Fig. 3:**
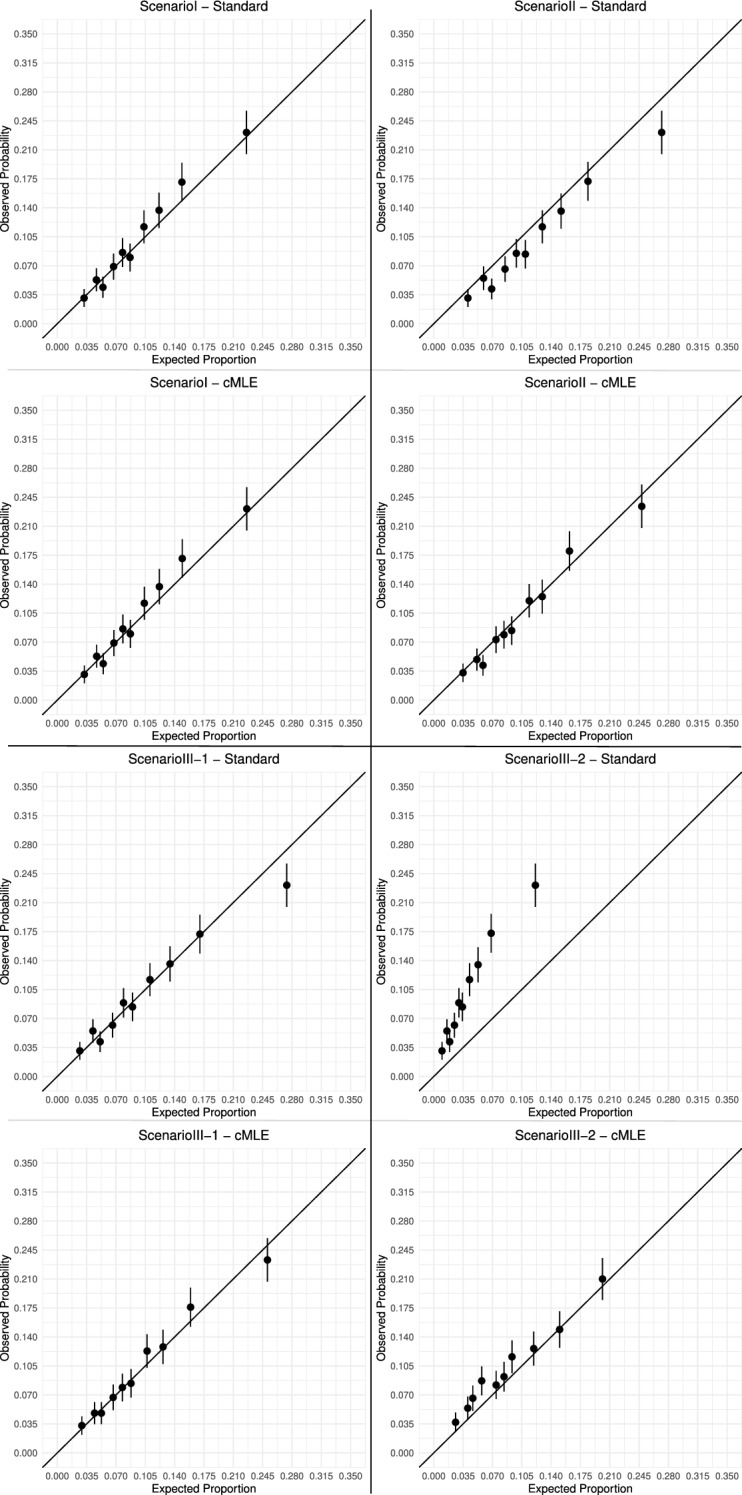
Model calibration using standard and cML methods

Figure 3 displays calibration plots for all three scenarios. The X-axis represents the “expected” proportion of cases calculated in risk intervals defined by the final fitted model, and Y-axis represents the observed probability of cases. The expected and observed numbers of cases agreed closely for the model fitted by the cML methods across all scenarios. In contrast, the model fitted by the Standard method over-predicted the risk in Scenario II across all risk levels, over-predicted in the high risk region in Scenario III with $a=0.5$ and $b=1.2$, and severely under-predicted the risk in Scenario III with $a=-0.5$ and $b=1.2$. These results showed that the proposed constraints effectively improved calibration, which was achieved through revising parameter estimates as shown in Tables 5 and 6.

**Table 5:** Estimation results under Scenarios I and II

	True	Scenario I							Scenario II						
		cML				Standard			cML				Standard		
		Est	Diff (%)	SE	ASE	Est	Diff(%)	SE	Est	Diff (%)	SE	ASE	Est	Diff (%)	SE
$\beta _0$	− 2.4	− 2.403	0	0.188	0.188	− 2.407	0	0.195	− 2.436	2	0.182	0.166	− 2.151	− 10	0.242
$\beta _{X_1}$	− 0.5	− 0.498	0	0.071	0.070	− 0.500	0	0.090	− 0.460	9	0.054	0.049	− 0.502	0	0.094
$\beta _{X_2}$	0.4	0.393	− 2	0.114	0.111	0.394	− 2	0.117	0.446	12	0.117	0.111	0.400	0	0.121
$\beta _{X_3}$	0.3	0.298	− 1	0.130	0.126	0.299	0	0.133	0.358	19	0.100	0.097	0.299	0	0.110
$\beta _{X_4}$	0.65	0.652	0	0.136	0.134	0.653	0	0.145	0.686	6	0.131	0.128	0.642	− 1	0.144
$\beta _{Z^c}$	0.1	0.098	− 2	0.061	0.060	0.098	− 2	0.061	0.097	− 3	0.059	0.055	0.097	− 3	0.057
$\tau _{\text {mean}\_{0}}$	− 2	− 1.999	0	0.032	0.032	− 1.999	0	0.032	− 1.999	0	0.044	0.044	− 1.999	0	0.044
$\tau _{\text {mean}\_{X_1}}$	0.1	0.100	0	0.015	0.015	0.100	0	0.015	0.100	0	0.018	0.018	0.100	0	0.018
$\tau _{\text {mean}\_{X_2}}$	− 0.1	− 0.100	0	0.020	0.021	− 0.100	0	0.020	− 0.101	0	0.021	0.021	− 0.101	0	0.021
$\tau _{\text {mean}\_{X_3}}$	0.1	0.101	0	0.025	0.025	0.101	0	0.025	0.100	0	0.021	0.021	0.100	0	0.021
$\tau _{\text {mean}\_{X_4}}$	0.1	0.100	0	0.031	0.032	0.100	0	0.031	0.100	0	0.031	0.031	0.100	0	0.031
$\sigma$	0.6	0.599	0	0.010	0.010	0.599	0	0.010	0.600	0	0.010	0.010	0.600	0	0.010

“True”: true parameter values in the target population; “Est”: mean estimates; “Diff (%)”: (Est-True)/True; “SE”: empirical standard error estimates; “ASE”: mean asymptotic standard error estimates

**Table 6:** Estimation results under Scenario III

	True	Scenario III $(a=0.5, b=1.2)$							Scenario III $(a=-0.5, b=1.2)$						
		cML				Standard			cML				Standard		
		Est	Diff (%)	SE	ASE	Est	Diff (%)	SE	Est	Diff (%)	SE	ASE	Est	Diff (%)	SE
$\beta _0$	− 2.4	− 2.450	2	0.210	0.197	− 2.386	− 1	0.262	− 1.899	− 20	0.182	0.202	− 3.399	42	0.397
$\beta _{X_1}$	− 0.5	− 0.537	7	0.067	0.061	− 0.603	21	0.102	− 0.683	37	0.050	0.056	− 0.603	21	0.152
$\beta _{X_2}$	0.4	0.452	13	0.125	0.123	0.483	21	0.133	0.148	− 63	0.125	0.129	0.481	20	0.202
$\beta _{X_3}$	0.3	0.346	15	0.111	0.109	0.359	20	0.118	− 0.107	− 136	0.133	0.149	0.356	19	0.179
$\beta _{X_4}$	0.65	0.724	11	0.140	0.141	0.770	18	0.152	0.459	− 29	0.161	0.171	0.766	18	0.220
$\beta _{Z^c}$	0.1	0.118	18	0.061	0.059	0.119	19	0.061	0.109	9	0.071	0.088	0.113	13	0.092
$\tau _{\text {mean}\_{0}}$	− 2	− 1.999	0	0.044	0.044	− 1.999	0	0.044	− 1.999	0	0.044	0.044	− 1.999	0	0.044
$\tau _{\text {mean}\_{X_1}}$	0.1	0.100	0	0.018	0.018	0.100	0	0.018	0.100	0	0.018	0.018	0.100	0	0.018
$\tau _{\text {mean}\_{X_2}}$	− 0.1	− 0.101	0	0.021	0.021	− 0.101	0	0.021	− 0.101	0	0.021	0.021	− 0.101	0	0.021
$\tau _{\text {mean}\_{X_3}}$	0.1	0.100	0	0.021	0.021	0.100	0	0.021	0.100	0	0.021	0.021	0.100	0	0.021
$\tau _{\text {mean}\_{X_4}}$	0.1	0.100	0	0.031	0.031	0.100	0	0.031	0.100	0	0.031	0.031	0.100	0	0.031
$\sigma$	0.6	0.600	0	0.010	0.010	0.600	0	0.010	0.600	0	0.010	0.010	0.600	0	0.010

“True”: true parameter values; “Est”: mean estimates; Diff (%): (Est-True)/True; “SE”: empirical standard error estimates; “ASE”: mean asymptotic standard error estimates

## Discussion

To build a new model upon an existing one by incorporating new predictors, the proposed constrained maximum likelihood estimation effectively enforces the new model to use the existing model as the “template” for prediction. The calibration of the new model is assured during model development when the existing one is well-calibrated. We assume that the distribution of conventional risk predictors is known in the target population, and impose a parametric model for the new predictors conditional on the conventional predictors. Then in the absence of an independent dataset from the target population, the utility of the new predictors can be more assessed. Importantly, our method does not require either the new or existing model perfectly capture the true relationship between the outcome and predictors, nor does it require an explicit functional form for the existing model. When the model is intended to be used for identifying population subgroups who have high risk, model calibration can be specifically emphasized in these interest risk regions through appropriate construction of constraints. Looser constraints can then be used in moderate risk regions to allow data to better inform model building. The effectiveness of such flexibility was demonstrated in numerical studies.

A question remains for our method is to what extent the data distribution for the source and target populations can differ. Theoretically, this was quantified by the concept of the feasible region, which contains the limit of cML estimates by Theorem 1. Practically, the source data that has a distribution more similar to that in the target population allows more informative model building. In the analysis of Penn Biobank data (Table 1), the subsample differed more from the NHIS than the full dataset. Comparing $\hbox {cML}^1$ using the two sets of data, Nbiops ($X_3$) and Numrel ($X_4$) were significant by Wald test in the full sample, but only Numrel remained significant in the subsample. With $\hbox {cML}^2$, Ageflb ($X_1$), Nbiops and Numrel were significant in the full data, but only Nbiops and Numrel remained significant in the subsample. We interpret the loss of significance of some variables in the subsample as information loss due to larger difference between the source and target populations.

The proposed approach requires a parametric model for the new predictors which is assumed identical in the source and target populations. This necessity arises when no information is assumed available on the new predictors in the target population, so the relationship between the new and standard predictors needs to be inferred from the study data. Mis-specification of this model may negatively affect the calibration of the new model. Parametric modeling for multiple predictors is generally challenging, and it is largely infeasible to consider nonparametric distribution due to the curse of dimensionality. It may be plausible to adopt more flexible model forms. When the new predictors are newly identified biomarkers, the relationship between the new and standard predictors may have already been studied at the discovery stage. Such prior information can then be incorporated into the proposed constraints. It is straightforward to adapt our method along this line.

## Proof of theoretical results

**Proof of Theorem** 1.

### Proof

Let $\hat{\varvec{\theta }}$ be the solution to the optimization problem (4). That is

$$\begin{aligned} \widehat{\varvec{\theta }}=\text{ argmax}_{\varvec{\theta }\in \varvec{\Theta }_{C}} N^{-1}l\left( \varvec{\theta }\right) . \end{aligned}$$

According to the law of large numbers, it is known that $N^{-1}l\left( \varvec{\theta }\right)$ converges in probability to $E_{S}\left\{ N^{-1}\left( \varvec{\theta }\right) \right\}$. Then in $\varvec{\Theta }_C$, we have

$$\begin{aligned} \sup \limits _{\varvec{\theta }\in \varvec{\Theta }_{C}}\left| N^{-1}l\left( \varvec{\theta }\right) -E_{S}\left\{ N^{-1}l\left( \varvec{\theta }\right) \right\} \right| \le \sup \limits _{\varvec{\theta }\in \varvec{\Theta }}\left| N^{-1}l\left( \varvec{\theta }\right) -E_{S}\left\{ N^{-1}l\left( \varvec{\theta }\right) \right\} \right| {\mathop {\rightarrow }\limits ^{P}} 0. \end{aligned}$$

Hence, applying Theorem 5.7 in Van der Vaart AW (2000), we have

$$\begin{aligned} \widehat{\varvec{\theta }}{\mathop {\rightarrow }\limits ^{P}}\varvec{\theta }^{*}. \end{aligned}$$

$$\square$$

**Proof of Theorem** 2

### Proof

The inequality constraints can be treated as equality constraints with the introduction of “slack” parameters. Let $\varvec{\xi }=\left( \xi _{1}^{+},\ldots ,\xi _{I}^{+},\xi _{1}^{-},\ldots ,\xi _{I}^{-}\right) ^T$ be a comfortable vector of slack parameters, and $\varvec{\eta }=\left( \varvec{\theta }^T,\varvec{\xi }^T\right) ^T\in \mathbb {R}^{1+p+q+2I}$. Denote

$$\begin{aligned}&F^+_{r}\left( \varvec{\eta }\right) \\&\ \ \equiv \frac{\int _{a_{r}< \varphi \left( {\textbf{x}}\right) \le b_{r}} \int _{{\textbf{z}}^{\prime }} P_{\varvec{\beta }}(Y=1|\varvec{X}={\textbf{x}},\varvec{Z}={\textbf{z}}')\delta \left( {\textbf{x}}\right) f_{\varvec{\tau }}\left( {\textbf{z}}^{\prime }\mid {\textbf{x}}\right) d{\textbf{z}}^\prime d{\textbf{x}}}{\int _{a_{r}<\varphi \left( {\textbf{x}}\right) \le b_{r}}\delta \left( {\textbf{x}}\right) d{\textbf{x}}}-(1+d_r) P^e_{r} +{\xi _{r}^+}^2, \end{aligned}$$

and

$$\begin{aligned}&F^-_{r}\left( \varvec{\eta }\right) \\&\ \ \equiv (1-d_r) P^e_{r} -\frac{\int _{a_{r}<\varphi \left( {\textbf{x}}\right) \le b_{r}} \int _{{\textbf{z}}^{\prime }} P_{\varvec{\beta }}(Y=1|\varvec{X}={\textbf{x}},\varvec{Z}={\textbf{z}}')\delta \left( {\textbf{x}}\right) f_{\varvec{\tau }}\left( {\textbf{z}}^{\prime }\mid {\textbf{x}}\right) d{\textbf{z}}^\prime d{\textbf{x}}}{\int _{a_{r}<\varphi \left( {\textbf{x}}\right) \le b_{r}}\delta \left( {\textbf{x}}\right) d{\textbf{x}}} +{\xi _{r}^-}^2, \end{aligned}$$

$r=1,\ldots ,I$. We can replace inequality constraints (4) with equality constraints (Luenberger et al. 1984; Boyd et al. 2004) and consider the equivalent optimization problem, minimizing

9 $$\begin{aligned} \begin{aligned}&l(\varvec{\beta },\varvec{\tau })=\sum _{i=1}^{N}\log \left[ \frac{\exp \left\{ Y_{i}\left( \beta _0+\varvec{\beta }_{{\textbf{x}}}^{T}\textbf{X}_{i}+\varvec{\beta }_{{\textbf{z}}}^{T}\textbf{Z}_{i}\right) \right\} }{1+\exp \left( \beta _0+\varvec{\beta }_{{\textbf{x}}}^{T}\textbf{X}_{i}+\varvec{\beta }_{{\textbf{z}}}^{T}\textbf{Z}_{i}\right) }f_{\varvec{\tau }}\left( \textbf{Z}_{i}\mid \textbf{X}_{i}\right) \right] \\&\text {subject~to}~~~F^+_{r}\left( \varvec{\eta }\right) =0;~~~F^-_{r}\left( \varvec{\eta }\right) =0,~~~ r=1,...,I. \end{aligned} \end{aligned}$$

Define $l\left( \varvec{\eta }\right) :=l\left( \varvec{\theta }\right)$, and $\varvec{F}\left( \varvec{\eta }\right) =\left( F_{1}^+\left( \varvec{\eta }\right) ,\ldots ,F_{I}^+\left( \varvec{\eta }\right) , F_{1}^-\left( \varvec{\eta }\right) ,\ldots ,F_{I}^-\left( \varvec{\eta }\right) \right) ^{T}$ be the equality constraints. The constrained maximization problem discussed above can be concisely and equivalently written as maximizing, with respect to $\varvec{\eta }$,

$$\begin{aligned} l\left( \varvec{\eta }\right) \ \text {subject to}\ \varvec{F} \left( \varvec{\eta }\right) =\varvec{0}. \end{aligned}$$

According to the existence and uniqueness of $\varvec{\theta }^{*}$, there exists a unique $\varvec{\eta }^{*} = \left( \varvec{\theta }^{*T},\varvec{\xi }^{*T}\right) ^{T}$ such that $\varvec{F} \left( \varvec{\eta }^{*}\right) =\varvec{0}$, and

$$\begin{aligned} E\left\{ N^{-1}l\left( \varvec{\eta }^{*}\right) \right\} =\max \limits _{\varvec{\eta }:\varvec{F}\left( \varvec{\eta }\right) =\varvec{0}}E\left\{ N^{-1}l\left( \varvec{\eta }\right) \right\} . \end{aligned}$$

The corresponding Lagrangian function is

$$\begin{aligned} l\left( \varvec{\eta }\right) +\varvec{F}\left( \varvec{\eta }\right) ^{T}\varvec{\lambda }, \end{aligned}$$

where $\varvec{\lambda }$ is the set of Langrage multipliers. Based on the KKT conditions, we obtain $\widehat{\varvec{\eta }}_{n}$ by solving the equation

10 $$\begin{aligned} \frac{\partial l\left( \widehat{\varvec{\eta }}\right) }{\partial \varvec{\eta }}+\left\{ \frac{\partial \varvec{F} \left( \widehat{\varvec{\eta }}\right) }{\partial \varvec{\eta }}\right\} ^{T}\varvec{\lambda }=\varvec{0}, \end{aligned}$$

where $\partial l\left( \widehat{\varvec{\eta }}\right) /\partial \varvec{\eta }\in \mathbb {R}^{\left| \varvec{\eta }\right| }$, $\partial \varvec{F}\left( \widehat{\varvec{\eta }}\right) ^{T}/ \partial \varvec{\eta }\in \mathbb {R}^{\left| \varvec{\eta }\right| \times \left| \varvec{F}\right| }$, $\left| \varvec{\eta }\right|$ is the length of $\varvec{\eta }$ and $\left| \varvec{F}\right|$ is the number of equality constraints. Indeed, (10) implies that $\partial l\left( \hat{\varvec{\eta }}\right) /\partial \varvec{\eta }$ is in the column space of $\partial \varvec{F}\left( \hat{\varvec{\eta }}\right) ^{T}/\partial \varvec{\eta }$. Thus, as long as $\left| \varvec{\eta }\right| >\left| \varvec{F}\right|$, we have a differentiable function $\textbf{U}\left( \varvec{\eta }\right) :\mathbb {R}^{\left| \varvec{\eta }\right| }\rightarrow \mathbb {R}^{\left| \varvec{\eta }\right| \times \left( \left| \varvec{\eta }\right| -\left| \varvec{F}\right| \right) }$, such that $\textbf{U}^{T}\left( \varvec{\eta }\right) \partial \varvec{F}\left( \varvec{\eta }\right) ^{T}/\partial \varvec{\eta }=\varvec{0}$, $\textbf{U}^{T}\left( \varvec{\eta }\right) \textbf{U}\left( \varvec{\eta }\right) =\varvec{I}$ for any $\varvec{\eta }$. And we will automatically have

11 $$\begin{aligned} \textbf{U}\left( \hat{\varvec{\eta }}\right) ^T\frac{\partial l\left( \hat{\varvec{\eta }}\right) }{\partial \varvec{\eta }}=\varvec{0}. \end{aligned}$$

For sufficiently large *N*, taking Taylor expansion of (11) about $\hat{\varvec{\eta }}$ at $\varvec{\eta }^{*}$gives us

12 $$\begin{aligned} \varvec{0}=&\textbf{U}^T\left( \hat{\varvec{\eta }}\right) \left[ \frac{1}{\sqrt{N}}\frac{\partial l\left( \varvec{\eta }^{*}\right) }{\partial \varvec{\eta }^{T}}+\frac{1}{N}\frac{\partial ^{2}l\left( \varvec{\eta }^{*}\right) }{\partial \varvec{\eta }\partial \varvec{\eta }^{T}}\sqrt{N}\left( \hat{\varvec{\eta }}-\varvec{\eta }^{*}\right) + O_{p}\left\{ \sqrt{N}\left\Vert \hat{\varvec{\eta }} -\varvec{\eta }^{*}\right\Vert ^{2} \right\} \right] \nonumber \\ =&\textbf{U}^T \left( \hat{\varvec{\eta }}\right) \frac{1}{\sqrt{N}}\frac{\partial l\left( \varvec{\eta }^{*}\right) }{\partial \varvec{\eta }^{T}} + \textbf{U}\left( \hat{\varvec{\eta }}\right) ^T \frac{1}{N}\frac{\partial ^{2}l\left( \varvec{\eta }^{*}\right) }{\partial \varvec{\eta }\partial \varvec{\eta }^{T}}\sqrt{N}\left( \hat{\varvec{\eta }}-\varvec{\eta }^{*}\right) + o_{p}\left\{ \sqrt{N}\left( \hat{\varvec{\eta }}-\varvec{\eta }^{*}\right) \right\} \nonumber \\ 
=&\textbf{U}^T\left( \hat{\varvec{\eta }}\right) \frac{1}{\sqrt{N}}\frac{\partial l\left( \varvec{\eta }^{*}\right) }{\partial \varvec{\eta }^{T}}+\left\{ \textbf{U}^T\left( \varvec{\eta }^{*}\right) +\left( \hat{\varvec{\eta }}-\varvec{\eta }^{*}\right) ^{T}\frac{\partial \textbf{U}^T\left( \varvec{\eta }^{*}\right) }{\partial \varvec{\eta }}\right\} \frac{1}{N} \frac{\partial ^{2}l\left( \varvec{\eta }^{*}\right) }{\partial \varvec{\eta }\partial \varvec{\eta }^{T}}\sqrt{N}\left( \hat{\varvec{\eta }}-\varvec{\eta }^{*}\right) \nonumber \\&+o_{p}\left\{ \sqrt{N}\left( \hat{\varvec{\eta }} -\varvec{\eta }^{*}\right) \right\} \nonumber \\ =&\textbf{U}^{T}\left( \hat{\varvec{\eta }}\right) \frac{1}{\sqrt{N}}\frac{\partial l\left( \varvec{\eta }^{*}\right) }{\partial \varvec{\eta }^{T}}+\textbf{U}^{T}\left( \varvec{\eta }^{*}\right) \frac{1}{N}\frac{\partial ^{2}l\left( \varvec{\eta }^{*}\right) }{\partial \varvec{\eta }\partial \varvec{\eta }^{T}}\sqrt{N}\left( \hat{\varvec{\eta }}-\varvec{\eta }^{*}\right) + o_{p}\left\{ \sqrt{N}\left( \hat{\varvec{\eta }}-\varvec{\eta }^{*}\right) \right\} . \end{aligned}$$

Following the theories in Crowder (1984); Stoica and CN (1998); Moore et al. (2008), let $\psi \left( t\right) :\mathbb {R}\rightarrow \mathbb {R}^{\left| \varvec{\eta }\right| }$ be a continuous differentiable map representing the feasible arc and $\psi \left( 0\right) =\varvec{\eta }^{*}$, $\psi \left( 1/N\right) =\hat{\varvec{\eta }}$ for any *N*. Then we have

$$\begin{aligned} \hat{\varvec{\eta }}-\varvec{\eta }^{*}=\psi \left( 1/N\right) -\psi \left( 0\right) =\frac{1}{n}\left. \frac{d\psi \left( t\right) }{dt}\right| _{t=1/n^{\prime }}, \end{aligned}$$

for some $0<1/n^{\prime }<1/N$. Note that $\textbf{C}\left\{ \psi \left( t\right) \right\} =0$ for all *t*. Therefore $\textbf{C}\left\{ \psi \left( 1/n^{\prime }\right) \right\} =0$ and

$$\begin{aligned} 0=\left. \frac{\partial \textbf{C}\left\{ \psi \left( t\right) \right\} }{\partial t}\right| _{t=1/n^{\prime }}=\left. \frac{\partial \textbf{C}\left\{ \psi \left( t\right) \right\} }{\partial \psi }\frac{d\psi \left( t\right) }{dt}\right| _{t=1/n^{\prime }}. \end{aligned}$$

This implies that $d\psi \left( 1/n^{\prime }\right) /dt$ isin the column space of $\textbf{U}\left( \varvec{\eta }^{\prime }\right)$, where $\varvec{\eta }^{\prime } = \psi \left( 1/n^{\prime }\right)$, i.e., $d\psi \left( 1/n^{\prime }\right) /dt = \textbf{U}\left( \varvec{\eta }^{\prime }\right) \varvec{Q}_N$ for some $\varvec{Q}_N\in \mathbb {R}^{\left| \varvec{\eta }\right| -\left| \varvec{F}\right| }$. Hence

13 $$\begin{aligned} \hat{\varvec{\eta }}-\varvec{\eta }^{*}=\frac{1}{N} \textbf{U}\left( \varvec{\eta }^{\prime }\right) \varvec{Q}_N. \end{aligned}$$

Inserting (13) into (12), we have

$$\begin{aligned} \varvec{0}=&\textbf{U}^{T}\left( \hat{\varvec{\eta }}\right) \frac{1}{\sqrt{N}}\frac{\partial l\left( \varvec{\eta }^{*}\right) }{\partial \varvec{\eta }^{T}}+\frac{1}{N}\textbf{U}^{T}\left( \varvec{\eta }^{*}\right) \frac{1}{N} \frac{\partial ^{2}l \left( \varvec{\eta }^{*}\right) }{\partial \varvec{\eta }\partial \varvec{\eta }^{T}} \sqrt{N}\textbf{U}\left( \varvec{\eta }^{\prime }\right) \varvec{Q}_{N}\\&+o_{p}\left\{ \sqrt{N}\left( \hat{\varvec{\eta }}-\varvec{\eta }^{*}\right) \right\} . \end{aligned}$$

Thus

14 $$\begin{aligned} \varvec{Q}_{N}=&\left\{ -\frac{1}{\sqrt{N}} \textbf{U}^{T} \left( \varvec{\eta }^{*}\right) \frac{\partial ^{2}\frac{1}{N}l\left( \varvec{\eta }^{*}\right) }{\partial \varvec{\eta }\partial \varvec{\eta }^{T}}\textbf{U}\left( \varvec{\eta }^{\prime }\right) \right\} ^{-}\textbf{U}^{T}\left( \hat{\varvec{\eta }}\right) \frac{1}{\sqrt{N}}\frac{\partial l\left( \varvec{\eta }^{*}\right) }{\partial \varvec{\eta }^{T}} \nonumber \\&+o_{p}\left\{ N\left( \hat{\varvec{\eta }}-\varvec{\eta }^{*}\right) \right\} , \end{aligned}$$

where $A^{-}$ denotes the Moore-Penrose inverse of matrix *A*. Combining (13) and (14), we have

$$\begin{aligned} \sqrt{N}\left( \hat{\varvec{\eta }}-\varvec{\eta }^{*}\right) =&\textbf{U}\left( \varvec{\eta }^{\prime }\right) \left\{ - \frac{1}{\sqrt{N }} \textbf{U}^{T}\left( \varvec{\eta }^{*}\right) \frac{\partial ^{2}\frac{1}{N} l\left( \varvec{\eta }^{*}\right) }{\partial \varvec{\eta }\partial \varvec{\eta }^{T}} \textbf{U}\left( \varvec{\eta }^{\prime }\right) \right\} ^{-}\textbf{U}^{T}\left( \hat{\varvec{\eta }}\right) \frac{1}{\sqrt{N}}\frac{\partial l\left( \varvec{\eta }^{*}\right) }{\partial \varvec{\eta }^{T}}\\&+o_{p}\left\{ \sqrt{N}\left( \hat{\varvec{\eta }}-\varvec{\eta }^{*}\right) \right\} . \end{aligned}$$

When $N\rightarrow \infty$, we have $\textbf{U}\left( \hat{\varvec{\eta }}\right) \rightarrow \textbf{U}\left( \varvec{\eta }^{*}\right)$ and $\textbf{U}\left( \varvec{\eta }^{\prime }\right) \rightarrow \textbf{U}\left\{ \psi \left( 0\right) \right\} =\textbf{U}\left( \varvec{\eta }^{*}\right)$ by consistency of $\hat{\varvec{\eta }}$ and continuity of $\textbf{U}$. Further, $N^{-1/2}\partial l\left( \varvec{\eta }^{*}\right) /\partial \varvec{\eta }\rightarrow N\left\{ \varvec{0},\tilde{\mathcal {I}}\left( \varvec{\eta }^{*}\right) \right\}$ in distribution by the central limit theorem, where $\tilde{\mathcal {I}}\left( \varvec{\eta }^{*}\right) \equiv E_{S}\left[ \left\{ \partial l_{1}\left( \varvec{\eta }^{*}\right) /\partial \varvec{\eta }\right\} ^{\otimes 2}\right]$, and we use $l_{1}\left( \varvec{\eta }^{*}\right)$ to denote the first summand in $l\left( \varvec{\eta }^{*}\right)$. Thus, $\sqrt{N}\left( \hat{\varvec{\eta }} -\varvec{\eta }^{*}\right)$ converges to a normal distribution with mean zero and variance ${\tilde{V}}\left( \varvec{\eta }^*\right) \tilde{\mathcal {I}}\left( \varvec{\eta }^*\right) {\tilde{V}}\left( \varvec{\eta }^*\right) ^{T}$, where

$$\begin{aligned} {\tilde{V}}\left( \varvec{\eta }^*\right) =\textbf{U}\left( \varvec{\eta }^*\right) \left[ \textbf{U}^T\left( \varvec{\eta }^*\right) E\{\frac{\partial ^2 l\left( \varvec{\eta }^*\right) }{\partial \varvec{\eta }\partial \varvec{\eta }^T}\}\textbf{U}\left( \varvec{\eta }\right) \right] ^{-}\textbf{U}^T\left( \varvec{\eta }^*\right) . \end{aligned}$$

Therefore, we know that $$\sqrt{N}\left( \hat{\varvec{\theta }}-\varvec{\theta }^{*}\right)$$ converges to a normal distribution with mean zero and variance $$A_{\left| \varvec{\theta }\right| }^T\left[ {\tilde{V}}\left( \varvec{\eta }^*\right) \tilde{\mathcal {I}}\left( \varvec{\eta }^*\right) {\tilde{V}}\left( \varvec{\eta }^*\right) ^{T}\right] A_{\left| \varvec{\theta }\right| 
}$$, where

$$\begin{aligned} A_{\left| \varvec{\theta }\right| }=\left[ \begin{array}{c} \varvec{I}_{\left| \varvec{\theta }\right| \times \left| \varvec{\theta }\right| } \\ \varvec{0}_{2I\times \left| \varvec{\theta }\right| } \end{array}\right] . \end{aligned}$$

Simple algebra calculation yields that

$$\begin{aligned} A_{\left| \varvec{\theta }\right| }^T\left\{ {\tilde{V}}\left( \varvec{\eta }^*\right) \tilde{\mathcal {I}}\left( \varvec{\eta }^*\right) {\tilde{V}}\left( \varvec{\eta }^*\right) ^{T}\right\} A_{\left| \varvec{\theta }\right| }=\textbf{V}\left( \varvec{\theta }^*\right) \mathcal {I}\left( \varvec{\theta }^*\right) \textbf{V}^{T}\left( \varvec{\theta }^*\right) . \end{aligned}$$

This completed the proof. $\square$

**Proof of Corollary** 1

### Proof

Under the assumptions in Corollary 1, we have

15 $$\begin{aligned} \left| g\left( \varvec{u}_{\text {new}}^{T}\widehat{\varvec{\beta }}\right) -g\left( \varvec{u}_{\text {new}}^{T}\varvec{\beta }^{*}\right) \right| \le M\left| \varvec{u}_{\text {new}}^{T}\left( \widehat{\varvec{\beta }}-\varvec{\beta }^{*}\right) \right| . \end{aligned}$$

Since $\varvec{u}_{\text {new}}$ is sub-Gaussian satisfying the conditions in corollary, we have

16 $$\begin{aligned} P\left( \left| \varvec{u}_{\text {new}}^{T}\left( \widehat{\varvec{\beta }}-\varvec{\beta }^{*}\right) \right| \ge t\mid \mathcal {D}\right) \le 2\exp \left\{ -t\sqrt{2}/\left( \left\Vert \widehat{\varvec{\beta }} -\varvec{\beta }^{*}\right\Vert \sigma _{\text {max}}\right) \right\} , \end{aligned}$$

where $\mathcal {D}$ is the data we used to estimate $\widehat{\varvec{\beta }}$. Combining (15) and (16), we have

$$\begin{aligned} P\left( \left| g\left( \varvec{u}_{\text {new}}^{T}\widehat{\varvec{\beta }}\right) -g\left( \varvec{u}_{\text {new}}^{T}\varvec{\beta }^{*}\right) \right| \ge tM\left\Vert \widehat{\varvec{\beta }}-\varvec{\beta }^{*}\right\Vert \sigma _{\text {max}}/\sqrt{2}\mid \mathcal {D}\right) \le 2e^{-t}. \end{aligned}$$

Hence,

$$\begin{aligned} g\left( \varvec{u}_{\text {new}}^{T}\widehat{\varvec{\beta }}\right) -g\left( \varvec{u}_{\text {new}}^{T}\varvec{\beta }^{*}\right) =O_{p}\left( \left\Vert \widehat{\varvec{\beta }}-\varvec{\beta }^{*}\right\Vert \right) . \end{aligned}$$

$$\square$$

## References

[CR1] Ankerst G, Gail M, Chatterjee N, Pfeiffer R (2016) Comparison of approaches for incorporating new information into existing risk prediction models. Stat Med 36(7):1134–56 27943382 10.1002/sim.7190 PMC8182952

[CR2] Bondy M, Lustbader E, Halabi S, Ross E, Vogel V (1994) Validation of a breast cancer risk assessment model in women with a positive family history. J Natl Cancer Inst 86:620–5 8003106 10.1093/jnci/86.8.620

[CR3] Boyd S, Boyd S, Vandenberghe L (2004) Convex optimization. Cambridge University Press, Cambridge

[CR4] Chatterjee N, Chen Y, Maas P, Carroll R (2016) Constrained maximum likelihood estimation for model calibration using summary-level information from external big data sources. J Am Stat Assoc 111:107–17 27570323 10.1080/01621459.2015.1123157 PMC4994914

[CR5] Costantino J, Gail M, Pee D, Anderson S, Redmond C, Benichou J, Wieand H (1999) Validation studies for models projecting the risk of invasive and total breast cancer incidence. J Natl Cancer Inst 91:1541–8 10491430 10.1093/jnci/91.18.1541

[CR6] Crowder M (1984) On constrained maximum likelihood estimation with non-iid observations. Ann Inst Stat Math 36:239–49

[CR7] Dalton JE (2013) Flexible recalibration of binary clinical prediction models. Stat Med 32(2):282–9 22847754 10.1002/sim.5544

[CR8] Debray T, Vergouwe Y, Koffijberg H, Nieboer D, Steyerberg E, Moons G (2015) A new framework to enhance the interpretation of external validation studies of clinical prediction models. J Clin Epidemiol 68(3):279–289 25179855 10.1016/j.jclinepi.2014.06.018

[CR9] Deng L, Ding J, Liu Y, Wei C (2018) Regression analysis for the proportional hazards model with parameter constraints under case-cohort design. Comput Stat Data Anal 117:194–206

[CR10] Gail M, Brinton L, Byar D, Corle D, Green S, Schairer C, Mulvihill J (1989) Projecting individualized probabilities of developing breast cancer for white females who are being examined annually. J Natl Cancer Inst 81(24):1879–86 2593165 10.1093/jnci/81.24.1879

[CR11] Luenberger D, Ye Y et al (1984) Linear and nonlinear programming, vol 2. Springer, New York

[CR12] McCarthy A, Liu Y, Ehsan S, Guan Z, Liang J, Huang T, Hughes K, Semine A, Kontos D, Conant E et al (2021) Validation of breast cancer risk models by race/ethnicity, family history and molecular subtypes. Cancers 14(1):45 35008209 10.3390/cancers14010045 PMC8750569

[CR13] Moore T, Sadler B, Kozick R (2008) Maximum-likelihood estimation, the Cramer–Rao bound, and the method of scoring with parameter constraints. IEEE Trans Signal Process 56:895–908

[CR14] Nocedal J, Wright S (1999) Numerical optimization. Springer, New York

[CR15] Pal Choudhury P, Wilcox A, Brook M, Zhang Y, Ahearn T, Orr N, Coulson P, Schoemaker M, Jones M, Gail M et al (2020) Comparative validation of breast cancer risk prediction models and projections for future risk stratification. J Natl Cancer Inst 112(3):278–85 31165158 10.1093/jnci/djz113 PMC7073933

[CR16] Pfeiffer R, Chen Y, Gail M, Ankerst D (2022) Accommodating population differences when validating risk prediction models. Stat Med 41(24):4756–80 36224712 10.1002/sim.9447 PMC10510530

[CR17] Rockhill B, Spiegelman D, Byrne C, Hunter D, Colditz G (2001) Validation of the Gail et al. model of breast cancer risk prediction and implications for chemoprevention. J Natl Cancer Inst 93:358–66 11238697 10.1093/jnci/93.5.358

[CR18] Song M, Kraft P, Joshi A, Barrdahl M, Chatterjee N (2015) Testing calibration of risk models at extremes of disease risk. Biostatistics 16(1):143–54 25027274 10.1093/biostatistics/kxu034 PMC4263225

[CR19] Steyerberg E (2019) Clinical prediction models. Springer, Berlin

[CR20] Stoica P, Ng BC (1998) On the Cramer–Rao bound under parametric constraints. IEEE Signal Process Lett 5(7):177–9

[CR21] Vergouwe Y, Moons K, Steyerberg E (2010) External validity of risk models: use of benchmark values to disentangle a case-mix effect from incorrect coefficients. Am J Epidemiol 172(8):971–80 20807737 10.1093/aje/kwq223 PMC2984249

[CR22] Zhai Y, Han P (2022) Data integration with oracle use of external information from heterogeneous populations. J Comput Graph Stat 31:1001–12

[CR23] Zheng J, Zheng Y, Hsu L (2022) Re-calibrating pure risk integrating individual data from two-phase studies with external summary statistics. Biometrics 78(4):1515–29 34390251 10.1111/biom.13543 PMC8895713

[CR24] Zheng J, Zheng Y, Hsu L (2022) Risk projection for time-to-event outcome leveraging summary statistics with source individual-level data. J Am Stat Assoc 117:1–13 36687294 10.1080/01621459.2021.1895810 PMC9855229

[CR25] Van der Vaart AW (2000) Asymptotic Statistics. Cambridge University Press, Cambridge.

